# α-Latrotoxin Tetramers Spontaneously Form Two-Dimensional Crystals in Solution and Coordinated Multi-Pore Assemblies in Biological Membranes

**DOI:** 10.3390/toxins16060248

**Published:** 2024-05-27

**Authors:** Alexis Rohou, Edward P. Morris, Julia Makarova, Alexander G. Tonevitsky, Yuri A. Ushkaryov

**Affiliations:** 1 Division of Cell and Molecular Biology, Imperial College London, Exhibition Road, London SW7 2AZ, UK; rohoua@gene.com; 2 Division of Structural Biology, The Institute of Cancer Research (ICR), London SW7 3RP, UK; ed.morris@icr.ac.uk; 3 Faculty of Biology and Biotechnology, HSE University, 117997 Moscow, Russia; jmakarova@hse.ru; 4 Shemyakin-Ovchinnikov Institute of Bioorganic Chemistry RAS, 117997 Moscow, Russia; tonevitsky@mail.ru; 5 Medway School of Pharmacy, University of Kent, Chatham ME4 4TB, UK

**Keywords:** α-latrotoxin, cryo-electron microscopy, two-dimensional crystals, membrane pore formation

## Abstract

α-Latrotoxin (α-LTX) was found to form two-dimensional (2D) monolayer arrays in solution at relatively low concentrations (0.1 mg/mL), with the toxin tetramer constituting a unit cell. The crystals were imaged using cryogenic electron microscopy (cryoEM), and image analysis yielded a ~12 Å projection map. At this resolution, no major conformational changes between the crystalline and solution states of α-LTX tetramers were observed. Electrophysiological studies showed that, under the conditions of crystallization, α-LTX simultaneously formed multiple channels in biological membranes that displayed coordinated gating. Two types of channels with conductance levels of 120 and 208 pS were identified. Furthermore, we observed two distinct tetramer conformations of tetramers both when observed as monodisperse single particles and within the 2D crystals, with pore diameters of 11 and 13.5 Å, suggestive of a flickering pore in the middle of the tetramer, which may correspond to the two states of toxin channels with different conductance levels. We discuss the structural changes that occur in α-LTX tetramers in solution and propose a mechanism of α-LTX insertion into the membrane. The propensity of α-LTX tetramers to form 2D crystals may explain many features of α-LTX toxicology and suggest that other pore-forming toxins may also form arrays of channels to exert maximal toxic effect.

## 1. Introduction

α-Latrotoxin (α-LTX), the vertebrate-specific component of the black widow spider venom, is widely used in studies of secretion in neurons and other cells [1,2]. The toxin causes a massive release of neurotransmitters from nerve terminals of all types of neurons [1]. Initially, its main effect was linked to the influx of Ca^2+^ into nerve terminals [3], which is well known to induce the exocytosis of synaptic vesicles containing neurotransmitters (reviewed in [2]). However, while α-LTX indeed makes Ca^2+^-permeable pores in artificial lipid membranes [3,4,5], it does not readily form such pores in biological membranes [6,7] and requires specific receptors to stimulate neuronal cells [8]. Moreover, α-LTX is able to stimulate neurons even in the absence of Ca^2+^ in the medium [2,9,10,11]. An analysis of the toxin’s diverse actions [2] revealed that some of its effects can indeed be explained by the formation of ion channels in biological membranes. In addition, α-LTX stimulates its presynaptic receptors, leading to intracellular signaling and subsequent neurotransmitter exocytosis. Obviously, receptor-mediated actions of α-LTX are important for the understanding of neuronal activity and its regulation, but the presence of a toxin pore complicates any studies of intracellular signaling. Therefore, it is important to understand how the toxin forms pores in lipid membranes and how such pores can be blocked or prevented from forming.

When the first 3D structure of α-LTX was revealed [12], it helped in dissecting these diverse modes of the toxin’s action (a 3D reconstruction of an α-LTX monomer aligned to its domain structure is shown in Figure S1a). Using cryogenic electron microscopy (cryoEM), it was shown conclusively that α-LTX organizes into tetramers to form membrane pores [12]. Consistent with that, the toxin loses its pore-forming activity if it is unable to tetramerize [13,14]. In particular, tetramerization (and pore formation) was found to be Mg^2+^- or Ca^2+^-dependent and to be impaired by treating the toxin with EDTA [13]. Most interestingly, genetic manipulations showed that introducing a 4-amino acid insert between the toxin’s N-terminal domain and the ankyrin repeats (ARs) (arrow in Figure S1a) [15] disrupts toxin tetramerization and pore formation [16]. This mutant, termed LTX^N4C^, still retained the ability to stimulate neurotransmitter release via its receptors but lost its pore-mediated effects [14,17]. In addition, other α-LTX mutants [18] lacked the activity associated with toxin pores, possibly because the parts affected by these mutations are involved in toxin tetramerization. All these data suggest that, by perturbing toxin tetramers, it is possible to separate the pore-dependent and receptor-dependent effects of α-LTX on neuronal cells.

Nevertheless, a further investigation of α-LTX pore formation itself remains a very important task. An understanding of the molecular mechanisms by which a hydrophilic α-LTX protein pierces the hydrophobic lipid bilayer, forming a channel permeable to ions and small hydrophilic molecules, would improve our knowledge of the diverse group of pore-forming toxins. Recent high-resolution cryoEM studies [19] revealed the high-resolution structures of two toxins homologous to α-LTX: α-latrocrustotoxin, αLCT, and δ-latroinsectotoxin, δ-LIT. Consistent with a similar domain architecture [20] and high sequence similarity of these three toxins [21,22,23], they have very similar 3D structures [12,19]. α-LCT and δ-LIT are capable of organizing into oligomers from dimers to tetramers, the latter having a rotational C4 symmetry and are very similar to α-LTX tetramers. However, there are also significant differences (discussed in detail below). For instance, α-LCT tetramers have not been observed in that study [19]. Also, the shape of α-LCT monomers precludes their tetramerization without undergoing substantial conformational changes which have not been revealed [19]. Furthermore, despite observing the top views of the δ-LIT tetramers, these authors have not produced their 3D reconstruction. Thus, the previously published model of the α-LTX tetramer [12], despite its lower resolution than that achieved recently by Chen and co-authors [19] for monomers and dimers, has remained the only available 3D reconstruction of the toxin’s pore-forming species to date.

To better understand the molecular rearrangements that occur in α-LTX and presumably other latrotoxins during their oligomerization and membrane insertion, we used cryoEM and single-channel recordings to investigate the behavior of α-LTX under the conditions known to support its pore-forming action in a biological setting, specifically, in the presence of Mg^2+^ [13].

Here, we describe the results of this work, which clearly demonstrate the propensity of α-LTX tetramers to spontaneously associate into 2D crystalline arrays. We observed α-LTX forming arrays of multiple channels that insert precipitously into biological membranes containing α-LTX receptors. We also identified two conformers of α-LTX tetramers, both in solution and in the 2D crystals, with central pores of different diameter. By comparing the 3D structure of α-LTX in its soluble and putative membrane-anchored form, we propose distinct molecular rearrangements that allow the toxin to change its hydrophobicity and insert itself into the membrane.

## 2. Results

### 2.1. The 2D Crystallization of α-LTX

It was previously reported [13] that Mg^2+^ and Ca^2+^ promote both α-LTX tetramer formation and its pore-mediated effects in synaptic nerve terminals. Therefore, we used divalent cations to probe the mechanism of α-LTX oligomerization. Ca^2+^ had been used in α-LTX pore permeability studies at concentrations ranging from 2 to 44 mM [5,6,24]. However, due to strong effects of Ca^2+^ on living cells and its propensity to increase channel noise [24,25], preference was given to Mg^2+^, which permeates the toxin channel as well [4].

Initially, to delineate the best conditions of α-LTX oligomerization, the radioactively labeled toxin was incubated with 2–100 mM Mg^2+^ for a period between 10 min and 7 days. The state of the toxin’s oligomerization was assessed by low-speed centrifugation. The formation of large supramolecular complexes that were removed by centrifugation correlated with both toxin and cation concentrations. Increasing [Mg^2+^] and the time of incubation facilitated the association of α-LTX molecules, such that 100 mM Mg^2+^ caused practically a complete aggregation of ^125^I-α-LTX within a week (Figure S1b), while 5 mM EDTA largely preserved the toxin’s solubility. To obtain a reasonable number of toxin complexes but without an uncontrollable aggregation, in subsequent experiments, α-LTX was incubated with 10 mM Mg^2+^ only.

**Figure 1:**
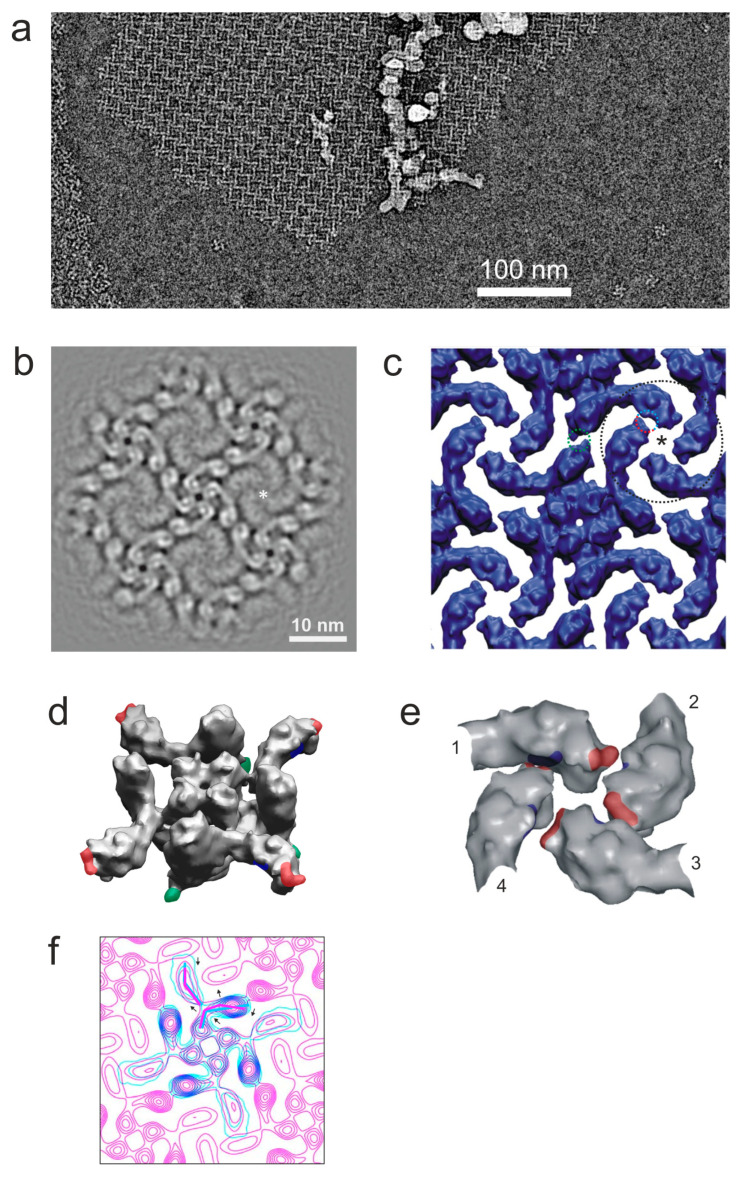
Two-dimensional crystals of α-LTX. (**a**) Crystals are formed at relatively low concentrations. The local concentration of toxin monomers was estimated to be 20 nM, by counting particles and assuming an ice thickness of 200 Å. Protein density is in white. (**b**) A symmetrized and bandpass-filtered (25 Å^−1^ to 5 Å^−1^) projection map obtained after the unbending and averaging of 192 unit cells of the α-LTX crystal. Asterisk: pseudo-four-fold cyclical symmetry point formed by four wing domains. The central pore of the tetramer is at the center of the image. (**c**) A 3D model of the 2D crystal obtained by fitting 3D models of α-LTX tetramers from [12]. Dotted colored circles mark the sites of inter-tetramer interactions; asterisk, pseudo-4-fold symmetry axis. (**d**) Potential regions of contacts between tetramers in the simulated 3D lattice: heterotypic interactions between wing domains (red and blue) and homotypic contacts between leg domains (green). (**e**) A detailed map of heterotypic “lock and key” interactions between four wings contributed by four tetramers (arbitrarily numbered 1-4 and corresponding to the area marked by a black circle in (**c**)). The numbers indicate adjacent tetramers participating in this 4-fold symmetry interaction. (**f**) Overlayed contour plots of a projection of the 3D reconstruction of the free α-LTX tetramer (cyan) (scaled and aligned to the 2D crystal map) and a projection map of the 2D crystal (pink) (as in (**b**); lowpass-filtered to ~20 Å^−1^). Arrows show conformational changes between the free tetramer and tetramer incorporated into the lattice, with the main shape of each tetramer indicated by broken lines of respective color.

The cryoEM of these samples revealed that Mg^2+^ treatment caused individual α-LTX tetramers to associate into flat and apparently rigid 2D sheets of variable extents (Figure 1a and Figure S1c,d). Such sheets appeared to be abundant if the toxin was not pretreated with EDTA, whereas almost no crystals were observed after the treatment of the venom with chelators, even if Mg^2+^ was added subsequently [13]. In relatively concentrated α-LTX solutions (6 μM), larger arrays were routinely observed after incubation on the EM grid for only a few seconds (Figure S1d). However, 2D crystals also occurred at low α-LTX concentrations (~20 nM, Figure 1a,b), suggesting that they resulted from specific tetramer–tetramer interactions, rather than concentration-dependent aggregation. Even though the largest arrays were approximately 0.5 by 0.5 μm and often filled the holes in the amorphous carbon film of the cryoEM grids entirely (~1 μm diameter), small crystalline patches often seemed more coherent. Such clusters were suggestive of a regular arrangement of the previously described toxin tetramers [12] into 2D crystals and seemed to grow by gradually acquiring loosely associated tetramers, with their subsequent tight packing into the grid (arrowheads and lines in Figure S1c). α-LTX tetramers have been reported to show a strong preference for interaction with air–water interfaces [12]. On the one hand, this indicates a hydrophobic character of the side with which the tetramer contacts the air. On the other hand, this interaction likely facilitates 2D crystallization by orienting the tetramers.

It is notable that some 2D crystal sheets had gaps in them, where one–four tetramers were missing (arrows in Figure S1c,d). Such holes were unlikely to be caused by the handling of the toxin solution during cryoEM sample preparation but instead were most probably created by an imperfect association of strings of tetramers during the rapid crystallization of the toxin (Figure S1c, right).

Diffraction patterns from such crystalline patches showed spots up to ~16 Å (Figure S1f) with a potential for medium-resolution analysis. An analysis of images by Fourier filtering and the unbending procedure [26] allowed us to build a projection map, which demonstrates that the 2D crystal is a square lattice of α-LTX tetramers, with parameters a = b = 14.7 nm and γ = 90° (Figure 1b), in which tetramers are arranged cyclically around pseudo-4-fold axes (asterisk in Figure 1b). Four wing domains, each from a different tetramer, come in close apposition around this axis (we follow domain nomenclature proposed previously [12]; see Figure S1a).

The resolution of this map was estimated to be ~12 Å (Figure S1g), and it reveals many internal features within the head and body domains. Strong elongated protein densities in the body domains, corresponding to the α-helices of the AR, are extended within the crystal plane but, due to the curvature of the body domain, are not individually resolved in the top view of the tetramer. The projection of each head domain contains one conspicuous spot of high protein density in the middle, wrapped by several elongated protein density spots. These structures correspond to the six α-helices of the Helical bundle domain (head domain in the earlier nomenclature [12]), a part of the recently published high-resolution structure of δ-LIT and α-LCT [19]. In the center of the Helical bundle domain is the hydrophobic H8 α-helix, which is roughly parallel to the symmetry axis of the tetramer and is surrounded by five other α-helices.

Interestingly, the resolution of the wing domains in 2D crystal images appeared to be markedly improved compared to average images of single-particle toxin tetramers (Figure 1b), probably because the multipoint interactions of the wings within the lattice (see below) restricted their movements. A comparison of the projection map patterns of this region with a simulated density map of ankyrin (PDB: 1N11) [27] suggested that the wing consists entirely of ARs and therefore can only contain the C-terminal end of α-LTX. This observation was also supported by the structures of α-LCT and δ-LIT [19], leading us to correct the earlier assignment of the α-LTX sequence with respect to its 3D and domain structures (Figure S1a).

To gain a better understanding of the 2D crystallization of the α-LTX tetramer, a virtual 3D lattice (Figure 1c) was built using the single-particle 3D reconstruction of the LTX tetramer produced by Orlova and co-authors [12]. Unit cell measurements from the image analysis of 2D arrays were taken as a starting point for a lattice building routine, which sought to optimize the distances between tetramers, their in-plane rotation and scaling, such that the vertical projection of the tetramers would best match the 2D map obtained from the analysis of the actual 2D lattice images. It was found that a lattice could be built without introducing overlaps between tetramers, which suggests that the changes in the structure of LTX induced by crystallization were only minor. Overall, there is a very good match of complementary surfaces, indicating that the tetramer structures are relatively rigid.

The resulting 3D model of the lattice identifies areas of contacts between tetramers (Figure 1c–e). Each monomer of each tetramer makes at least three contacts with its neighbors (colored patches in Figure 1d). The tip of each wing nestles into the concave side of a neighboring tetramer’s wing, and its concave side is itself in contact with the tip of another wing (Figure 1e). Apart from these heterotypic wing–wing contacts, which involve two distinct areas of the wing, homotypic inter-tetramer contacts appear to occur between the leg domains at the bottom of the body (green in Figure 1d), which is consistent with the idea that this area is hydrophobic (see Discussion). In fact, the interaction of large, unperturbed sheets of tetramer lattices with the water–air interface requires that all monomers in a toxin tetramer equally interact with a hydrophobic plane (air or lipid membrane).

However, a careful comparison of the crystal projection map (as in Figure 1b) with the symmetry axis projection of the 3D single-particle reconstruction [12] suggests that crystallization does cause slight but important conformational changes to the toxin monomers within each tetramer that occur within the crystal plane (Figure 1f). This requirement for the tetramers to undergo some conformational adjustments in order to fit into the 2D crystal is consistent with our observation of α-LTX tetramers interacting with 2D lattices before gradually taking up their position in them (Figure S1c).

One important feature of the 2D crystals is that all toxin tetramers in them are arranged unidirectionally, i.e., the head domains of all tetramers face one side of the plane of the lattice, and the bottom sides face the opposite plane. In addition, the holes at the centers of tetramers are perpendicular to the plane of the flat lattices. Given that the tetramer is the toxin’s pore-forming entity [12,13], and that the central hole likely forms the transmembrane channel, the structure of the 2D lattices suggested that α-LTX could simultaneously form a large number of pores in the membrane.

### 2.2. Formation of Pore Arrays in Biological Membranes

To assess the relevance of α-LTX crystallization to its biological function, we investigated the toxin’s ability to form ion channels in biological membranes under the conditions favoring 2D crystallization. To improve the toxin’s insertion into the cell membrane, we used human embryonic kidney (HEK293) cells expressing a high-affinity α-LTX receptor, ADGRL1 (latrophilin-1). Although the formation of α-LTX channels was reported in untransfected HEK293 cells [24], this required a very high toxin concentration unless the cells were transfected with ADGRL1. One explanation is that HEK293 cells normally express ADGRL2 [28,29], a homolog of ADGRL1 which has a 14-fold lower affinity for α-LTX than ADGRL1 [30], thus providing low-affinity binding sites for the toxin in untransfected cells. On the other hand, α-LTX forms similar channels in the cell membrane irrespective of which receptor is used to facilitate its insertion [24]. Therefore, to study α-LTX channel formation but avoid any potential intracellular signaling, we used HEK293 stably expressing a mutant receptor, which only contained the N-terminal domain of ADGRL1 and a single transmembrane domain [31]. When this construct is expressed in cultured mammalian cells, it provides strong binding sites for α-LTX but is unable to mediate any intracellular signals.

**Figure 2:**
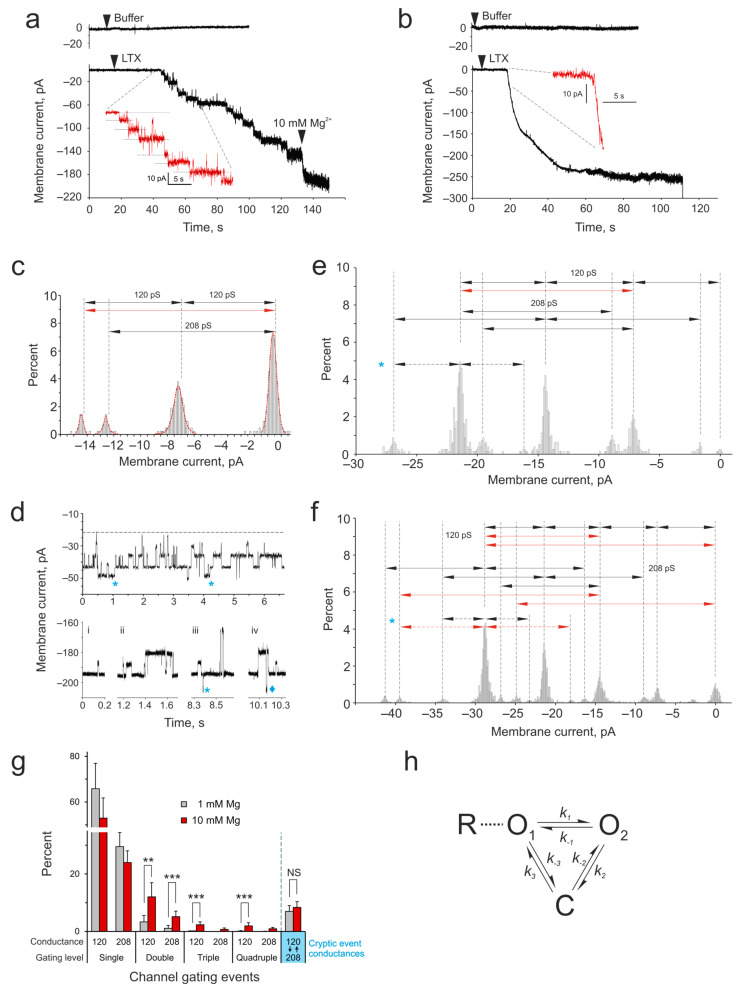
Properties of α-LTX channels formed in biological membranes. α-LTX pore formation in the membrane of HEK293 cells stably expressing ADGRL1. (**a**,**b**) Whole-cell current I_m_ was recorded in cells voltage-clamped at −60 mV. The cells were perfused with a Ca^2+^-free buffer containing Mg^2+^. Top traces, no changes in I_m_ were observed in cells without α-LTX addition. Bottom traces, α-LTX was preincubated with 1 mM (**a**) or 10 mM (**b**) Mg^2+^ for 20 min and applied close to the recorded cells (as indicated). Note that inward currents exceeding 200 pA appeared in the treated cells gradually (**a**) or abruptly (**b**). Insets, same traces scaled up around the time of channel formation. Dashed lines show current steps corresponding to the openings of individual toxin channels. In some experiments (*n* = 3) with low Mg^2+^ (**a**), the cells were additionally perfused with buffer containing 10 mM Mg^2+^ (as indicated). All results are representative of several independent experiments; *n* = 7–11 for each of the 4 experimental conditions. (**c**) The distribution of current amplitudes in all the recordings is as in A, bottom. The histogram shows channels with conductance levels of 120 ± 9 pS and 208 ± 11 pS. Gating events are indicated by arrows. A number of duplicate events can be identified (red arrow). (**d**) α-LTX channels in membrane patches behave independently or in coordination. Representative fragments of single-channel I_m_ recordings in outside-out membrane patches after channels were formed by α-LTX pretreated with 1 mM Mg^2+^ (top) or 10 mM Mg^2+^ (bottom). Blue asterisks, “cryptic” gating events; diamond, the simultaneous opening of 2 high-conductance channels. (**e**,**f**) Relative current amplitude distribution in 1 mM (**e**) and 10 mM (**f**) Mg^2+^, obtained from recordings as in d. A “0” membrane current is the lowest amount of inward current observed, although I_m_ never reaches 0 pA. Two channel states are apparent, with conductances of 120 and 208 pS. Arrows indicate gating events between channel states, identified visually in the recordings. Note duplicate (**e**,**f**) and quadruplicate (**f**) gating events (red arrows). Asterisks in (**d**–**f**) indicate the “cryptic” events with amplitudes of ~5.3 and ~10.6 pA. (**g**) A comparison of the frequency of single and simultaneous multiple gating events produced by the toxin treated with 1 mM or 10 mM Mg^2+^. The rightmost bars show the frequency of “cryptic” events, when channels transition between two conductance levels without closing. The data shown are the means ± *SD*; the results of statistical tests: *NS*, non-significant; **, *p* < 0.01; ***, *p* < 0.001. The number of independent experiments in (**d**–**g**), *n* = 23; representative results are shown. (**h**) A proposed model of α-LTX channel activity. O, two open states with distinct conductances; C, closed state; R, an α-LTX receptor.

To detect the formation of ion-permeable α-LTX pores in the cell membrane, whole-cell membrane currents (I_m_) were monitored in separately growing cells voltage-clamped at −60 mV (Figure 2). No significant current changes were recorded before α-LTX was added to the cells (Figure 2a,b, top traces). However, the application of the toxin, preincubated with 1 or 10 mM Mg^2+^, gave rise to macroscopic inward (negative-going) membrane currents (I_m_ reaching nA levels) in both conditions (Figure 2a,b, bottom traces). These currents developed gradually after toxin preincubation in low Mg^2+^ (Figure 2a) and, at least in the initial phase, showed clear stepwise increases (inset). These current steps with amplitudes of ~7.2 pA and ~12.5 pA were consistent with the consecutive formation (and gating) of multiple membrane channels with two unitary conductance levels, 120 ± 9 pS and 208 ± 11 pS in the ionic conditions employed (Figure 2c). These channels were apparently formed by individual toxin tetramers, which constitute a prevalent molecular species in 1 mM Mg^2+^ solutions. In contrast, after preincubation with 10 mM Mg^2+^ which stimulates 2D crystallization, the toxin produced extremely large currents that developed very quickly and with very few, if any, discernible steps (Figure 2b). Often these avalanche-like currents led to a loss of the patch due to the collapse of membrane resistance. Although α-LTX channels are permeable to Mg^2+^ [4], this difference in I_m_ between the two conditions studied could not be caused by the increase in Mg^2+^ concentration and a corresponding rise in the conductance of individual toxin channels, because the addition of 10 mM Mg^2+^ to cells recorded in 1 mM Mg^2+^ caused only a slight increase in the inward current (Figure 2a).

To test whether α-LTX channels in the cell membrane were organized into groups corresponding to 2D lattices, we investigated their behavior by the single-channel voltage-clamp technique in membrane patches excised from receptor-expressing HEK293 cells (Figure 2d–f). As expected, when Mg^2+^-pretreated toxin was added to the bath, it induced a large inward current in both cases. In low Mg^2+^, single-channel gating events with a variety of amplitudes were recorded (Figure 2d,e). A careful visual inspection of the recordings allowed us to identify channel transitions that corresponded to two main channel states, which were similar to those observed in the whole-cell experiments above (Figure 2a–c). These channels had conductance levels of 119.5 ± 8 pS and 208 ± 12 pS and behaved independently of each other. We also observed a small number of double events, where two channels of the same conductance opened simultaneously within our sampling interval of 100 μs (Figure 2e, red arrow). In contrast, the channels formed by the toxin in 10 mM Mg^2+^ behaved in a much more coordinated manner (Figure 2d,f): while these recordings also demonstrated individual gating events with conductances of ~120 pS and ~208 pS, a number of large-scale gating events were also observed, with current amplitudes up to ~30 pA (Figure 2f). Again, by carefully inspecting each recording, it was possible to assign I_m_ amplitude peaks to the gating of single or multiple channels with the same two conductance levels (but see a description of “cryptic” events below). Many of the events observed in 10 mM Mg^2+^ were consistent with a synchronous gating of groups of two to four channels (Figure 2(di–div); red arrows in Figure 2f).

We then compared the number of single and multiple simultaneous gating events under the two toxin treatment conditions. As Figure 2g shows, simultaneous (or unresolved) double events were much more prevalent when the toxin was treated with 10 mM Mg^2+^. Triple and quadruple gating events were observed under the toxin crystallization conditions only (10 mM Mg^2+^).

Interestingly, both in 1 mM and 10 mM Mg^2+^ membrane patch recordings, we also noticed events whose amplitudes (~5.3 and ~10.6 pA) did not correspond to the two heretofore identified main conductance levels of 120 and 208 pS (blue asterisks in Figure 2d–f). It is possible that these current changes reflected additional, rare channel substates with conductances of ~88 and ~176 pS. However, the observation of episodes when the closing of low-conductance channels (negative current decreased by ~7.2 pA) was followed by the opening of high-conductance channels (negative current increased by ~12.5 pA), which resulted in net current shifts of ~5.3 pA or ~10.6 pA (see an event marked with ♦ in Figure 2d), suggested that these “cryptic” events actually resulted from the direct (or temporally unresolved) channel transitions between the main low- and high-conductance states (120 and 208 pS), i.e., without intermediate closing. A proposed scheme of the gating of α-LTX channels, which have two open states and one closed state, is presented in Figure 2h. The number of such “cryptic” events was relatively low and did not depend on the toxin treatment conditions (Figure 2g), although double “cryptic” events (~10.6 pA) were only detected in 10 mM Mg^2+^ recordings.

We interpret these results as an indication that as α-LTX assembles into 2D crystals at the cell surface, these clusters make multiple membrane pores, with individual tetramers in the lattice incorporating into the membrane precipitously and subsequently opening and closing in synchrony.

### 2.3. Conformational Changes in the α-LTX Tetramer

Could the toxin molecule in solution adopt distinct conformations corresponding to the distinct open states of the channel and could such conformations be detected using cryoEM? The early 3D reconstruction [12] had a relatively modest resolution (~14 Å) and probably included tetramers with different conformations. To identify putative conformers within a similar dataset, an investigation into its heterogeneity was undertaken following a procedure like that described by Klaholz [32]. A subset of 20,000 particles was selected from micrographs and used to produce 2500 3D reconstructions, which were subjected to multivariate statistical analysis (MSA) (Figure S2a).

**Figure 3:**
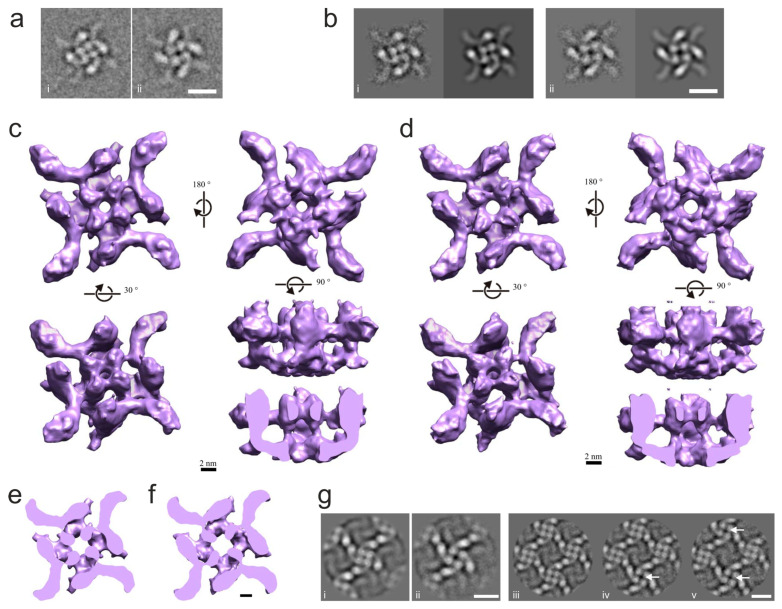
Two different conformers of the α-LTX tetramer have central channels of different size. (**a**) (**i**, **ii**). Two top view class averages identified visually in the dataset, with distinct conformations. Scale bar: 10 nm. (**b**) Two conformations of the toxin tetramers obtained by single particle analysis. Average representative images (**left**) and corresponding 3D reprojections (**right**) from refined “narrow-pore” (**i**) and “wide-pore” (**ii**) 3D reconstructions of the LTX tetramer. Scale bar: 10 nm. (**c**,**d**) Surface renderings of the “narrow-pore” (**c**) and the “wide-pore” (**d**) 3D reconstructions of the single-particle tetramer. Volumes are rendered so that the surface-enclosed volume corresponds to a molecular weight of 520 kDa. (**e**,**f**) Horizontal sections near the bottom junction of the head and body domains of the “narrow-pore” (**c**) and the “wide-pore” (**d**) 3D reconstructions. Note the larger diameter of the cavity in the center of the tetramer in the “wide” conformation. Scale bar, 25 nm. (**g**) An observation of two conformers in 2D crystals. (**i**, **ii**) Average images of unit cells, centered on individual tetramers in the “narrow-pore” (**i**) and “wide-pore” (**ii**) conformation. Note the close similarity between 2D-crystallized and soluble tetramers. (**iii**–**v**) Average images of the unit cells of the 2D crystal showing the following: (**iii**) all 4 tetramers in the “narrow” conformation, (**iv**) 3 tetramers in “narrow” and 1 in “wide” conformation and (**v**) 2 tetramers in “narrow” and 2 in “wide” conformation. “Wide-pore” conformers are indicated by arrows. The diagonal distribution of the two conformers in the quartet of tetramers was prevalent in 2D lattices. Scale bar: 10 nm.

The second eigenvolume (i.e., the one that accounts for the most variance among the 3D reconstructions; Figure S2(aii)) was particularly interesting, because it appeared to reflect variations we had observed in top-view class averages: some tetramers looked curled-up (Figure 3(ai)), while other tetramers seemed somewhat uncoiled (Figure 3(aii)). Most intriguingly, the apparent radius of the central pore was larger in the uncoiled reconstructions than in the curled-up ones. For this reason, the two types of reconstruction were termed “narrow-pore” and “wide-pore”. The selection of individual 3D reconstructions (from the set of 2500) on the basis of their second eigenvalue and visual examination (Figure S2b,c) clearly suggested a linear relationship between the second eigenvalue and the wide-pore aspect of a 3D reconstruction, with MSA providing a quantitative measure of conformational differences between the 3D reconstructions.

Using this approach enabled us to produce 3D reconstructions of two conformers of the toxin tetramer. Following 2D classification, 344 class averages (corresponding to 13,247 individual images) were separated by calculating their correlation with reprojections from reconstructions with the narrowest (Figure S2(ci)) and widest (Figure S2(cv)) pore, producing two groups of average images: a narrow-pore group of 216 averages (corresponding to 8354 images; example Figure 3(bi)) and a wide-pore group of 128 average images (corresponding to 4893 images; Figure 3(bii)). The iterative refinement of the two sets of projections led to two 3D reconstructions corresponding to the two conformational states (Figure 3c,d). The narrow-pore reconstruction (Figure 3c and Figure S2d, left), with a final resolution of 12.8 Å (Figure S2e, left), and the wide-pore reconstruction (Figure 3d and Figure S2d, right), with a final resolution of 14 Å (Figure S2e, right), were stable in angular reconstitution refinement, included a significant number of side views (i.e., were not strictly anchored at the air–water interface; Figure S2f) and displayed the differences expected from the 3D MSA experiment (Figure S2b).

The 3D reconstructions (Figure 3c,d), which overall are very similar to that in the earlier work [12], demonstrate a range of differences between the two conformers, with the most prominent being the size of the opening in the center of each tetramer type (top views in Figure 3c,d). Compared to the narrow-pore structure, the wide-pore conformer demonstrates the following in-plane changes to the domains within its monomers (Figure 3d,f): the contact between the wing and the head appears to be more extensive, and the shortening of the wing causes the heads to rotate slightly counterclockwise, appear slimmer and move away from the center of the tetramer. In addition, the body densities become uncoiled and also move slightly outwards. As a result, the size of the channel at its narrowest point increases from 11 Å in the narrow-pore conformer to 13.5 Å in the wide-pore structure.

These data demonstrated that the α-LTX tetramer can exist in at least two conformations that differ crucially by the diameter of the central channel. Given that the tetramer is the channel-forming entity of the toxin, and the central pore forms the ion-permeable channel, these conformers may correspond to the two types of toxin channels detected electrophysiologically with two characteristic conductance levels (Figure 2c–f). However, as the toxin also produces two types of channels (Figure 2f) under conditions favorable to oligomerization (high Mg^2+^), one would also expect to observe the two tetramer conformations in 2D crystals. To test this, we subjected the 852 unit cell images that had led to our projection map (Figure 1b) to MSA and classification and obtained ten class averages, of which six resembled the narrow-pore conformation, two the wide-pore conformation and two consisted of unit cells at the edges of the lattice. To probe this further, we reanalyzed raw images of 2D crystals using reprojections from our narrow-pore and wide-pore reconstructions as references and isolated images of unit cells within 2D crystals that corresponded to each conformation. Class average images obtained this way were essentially identical to either the narrow-pore or the wide-pore single particle images (compare Figure 3(bi,bii) with Figure 3(gi,gii)). However, the distribution of protein densities inside the heads of wide-pore tetramers became even more defined and consistent with the localization of α-helices in the Helical bundle domain of α-LCT and δLIT [19]. As expected from the fact that our initial projection map (Figure 1b) resembled the narrow-pore conformation, we found that narrow-pore conformers were more prevalent in the lattices (Figure 3(giii,giv)). In 2D class averages where two narrow and two wide tetramers were present, the wide-pore conformers usually occupied diagonal positions (Figure 3(gv)), suggesting that conformational changes may be constrained by the lattice.

These data demonstrate that α-LTX tetramers, both as individual soluble particles and as unit cells of 2D crystals, take up two different conformations that are characterized by different diameters of the central channel. Given that the toxin also forms two types of channels detected electrophysiologically, we hypothesize that the two tetramer conformations correspond to the two types of ion-permeable channels formed by α-LTX in the cell membrane.

### 2.4. Domain Arrangement in α-LTX

**Figure 4:**
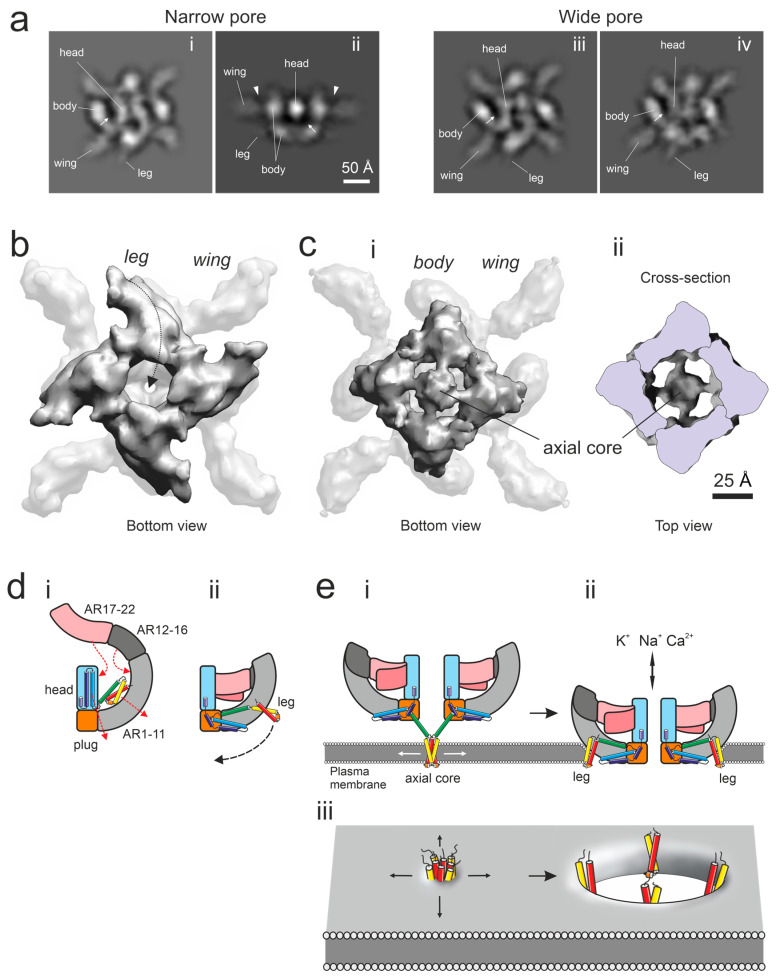
Conformational changes in the α-LTX tetramer and membrane insertion. (**a**) Selected reprojections of 3D reconstructions of soluble α-LTX tetramers in the narrow-pore (**i**, **ii**) and wide-pore (**iii**, **iv**) conformations. Note the following features: spots of higher protein density at the top of the body domains (**i**–**iv**) and at the bottom of the structure (**ii**); the top of body domains extending above the wing domains (arrowheads in (**ii**)); a clearly visible leg domain (**i**–**iv**) and the apparent lack of protein material in the space between the body and head domains (arrow). (**b**) The folding of the leg domains under AR1-5, as viewed from the bottom of the 3D reconstruction of the narrow-pore tetramer. The arrow shows a change in leg conformation prior to the membrane anchoring of the tetramer. (**c**) A 3D reconstruction in which the 4 leg domains are rearranged from the side of the body to the axial position. This tetrameric core domain serves as a membrane anchor and is viewed from the bottom of the 3D structure (**i**) and from within the cut-open tetramer (**ii**). (**d**) A scheme of conformational changes from the flat form (**i**) to the soluble form of the α-LTX monomer (**ii**) and its subsequent transformation leading to membrane anchoring. Note the changes in the distribution of α-helices H1-H7a, encoded by rainbow colors, starting from the N-terminal α-helix (H1, red). Yellow, the hydrophobic α-helix H3. (**e**) A scheme of the main stages of pore formation by α-LTX tetramers: anchoring in the membrane with the help of a core/proboscis formed by the leg domains (**i**) and the membrane insertion of the tetramer (**ii**). (**iii**), Hypothetical formation of a toroidal lipidic pore in the membrane by the leg domains which facilitates the insertion of the whole α-LTX tetramer into the membrane (**ii**).

While studying α-LTX tetramers as single particles and unit cells of 2D crystals, we compared our reconstructions to the recently determined high-resolution cryoEM structures of two related toxins from black widow spider venom, α-LCT and δ-LIT [19]. There was a high similarity in the shape of many domains. However, there were also certain conspicuous differences in structure details (Figure 4a; see also Section 3): (1) the wings in our reconstruction are attached below the top of the body domains (arrowheads in Figure 4(aii)) and contact the middle of the head domain (Figure 3c,d, while in α-LCT, the wing domain simply represents a bend at the top of the extended body domain and does not interact with the head at all [19]; (2) the top of the body domains in our model contains a higher protein density, consistent with the AR domain folding onto itself (Figure 4(aii,aiii)), while in α-LCT and δ-LIT, the body domain is not folded but meandering throughout its length; (3) there is no protein density in the space between the head and body domains in our model (arrows in Figure 4a), while in α-LCT and δ-LIT, this area contains a folded Connector domain [19] and (4) all our reconstructions invariably contain the leg domain attached to the bottom part of the body domain and protruding sideways (Figure 4(ai–aiv)), which is totally absent from α-LCT and δ-LIT reconstructions. The presence of the leg domain is especially obvious in the bottom views of our 3D tetramer reconstructions (Figure 4b), which demonstrate a large amount of protein material attached below the ARs at the bottom of the tetramer.

Furthermore, during the course of refinement, from a subset of raw images, we were able to produce a 3D reconstruction (Figure 4(ci)), which resembles the narrow-pore conformation but clearly differs from it by (1) lacking the leg domain in the lower part of the body and (2) containing an additional axial occlusion below the body domains. This formation, called an axial tetrameric core, is attached below the pore opening at the bottom of the tetramer (Figure 4(cii)). Due to its strong preference for the water–air interface, this structure was resolved at a lower resolution than our other 3D reconstructions, but it still indicates that the leg domains may be able to detach from the low body domains, swing under the tetramer (arrows in Figure 4(b,dii)) and form a tetrameric axial core, or proboscis, which occludes the central canal of the tetramer (schematically depicted in Figure 4(ei)). Based on computer modeling, this core contains α-helices H1-H3, including the hydrophobic H3, which may allow the core to anchor at the water surface and possibly in the membrane.

## 3. Discussion

The 2D crystals of α-LTX tetramers described here demonstrate a fascinating ability of the toxin to form large flat arrays under physiological conditions. In these crystals, each tetramer makes 3-point triangular contacts with each of its four neighbors, thus strengthening the rigidity of the crystal plane. In addition to participating in the lattice formation, the hydrophobic helices in the legs may enable the interaction of the lattice with the cell membrane. The simplest crystal assembly (nucleus) of four tetramers provides on its periphery the same binding points for other tetramers, which attach to the initial nucleus in the form of strings of tetramers of various length (Figure S1c). In this configuration, the central channel in the middle of each tetramer (formed by the four heads) appears perpendicular to the plane of the crystal and therefore makes channel formation in the membrane the geometrically most favorable.

Pore formation is characteristic of all studied members of the latrotoxin family, α-LTX, α-LCT, δ-LIT and αLIT, and has been widely described in the literature [6,7,19,23,24,33,34]. Since early publications on the structure and properties of α-LTX tetramers [12,13,16], it has been accepted that it is the tetramers of latrotoxins that form membrane channels. From this standpoint, the 2D crystals of α-LTX, despite their high propensity to assemble and their configuration conducive of forming multiple channels, could be an irrelevant curiosity, not involved in the toxicology of latrotoxins. On the other hand, the conditions under which we stimulated α-LTX crystallization (10 mM Mg^2+^) were very close to physiological. Furthermore, due to their high concentration in the venom gland, latrotoxins might form 2D crystals in spider venom. Indeed, very large molecular assemblies were observed when isolating α-LTX from the venom [13]. Also, it is possible that single latrotoxin tetramers can oligomerize under completely physiological conditions when they interact with the receptors and membrane lipids. This hypothesis is borne out by the fact that pore formation by individual α-LTX tetramers occurs as a chain reaction, where no channels are made for a long time, but once the first molecule inserts into the membrane, others follow in fast succession [7,16,24] (Figure 2b). Finally, under the conditions when toxin crystallization invariably occurs, we recorded coordinated arrays of α-LTX channels in the cell membrane (Figure 2d,f; see also below). These considerations strongly suggest that the 2D lattices of α-LTX tetramers are likely to be involved at least in some biological actions of this toxin.

One of the most interesting features of these 2D crystals is their relative overall rigidity, which guarantees their flatness and internal order, combined with some flexibility within the plane of the crystal, which allows tetramers to undergo conformational transitions and change the size of their central channel. As described under the Results, these conformational changes increase the channel diameter from ~11 Å to 13.5 Å, which is likely to increase the permeability of the channel. On the other hand, the crystallization apparently imposes some constraints on each unit cell and encourages coordinated/simultaneous conformational changes in several tetrameric channels (Figure 3(gv)), giving rise to the synchronous opening/closing of groups of channels.

Our physiological experiments further validate these findings. First, we observed that individual α-LTX tetramers insert into the cell membrane consecutively and form two types of ion channels with conductances of 120 and 208 pS (Figure 2a,c). While the initial steps of channel formation by crystallized α-LTX were very fast and not resolved in our recordings (Figure 2b), once the channels were formed, they also demonstrated two substates with the same two conductance levels (Figure 2d–f). These two conductance levels likely reflect the two conformations of α-LTX tetramers that we discovered in our work (Figure 3). Two conductance states have also previously been reported previously for α-LTX [6], α-LCT and δ-LIT [19], although specific channel conductances were different in all these publications, which might be due to the different ionic conditions employed.

Second, toxin channels in 2D crystals behaved in a significantly coordinated manner, when the simultaneous gating of two–four channels was observed (Figure 2e–g). It is possible that these synchronous events were in fact consecutive single gating steps, which occurred faster than our sampling interval of 100 μs, and so were not resolved in our recordings. On the other hand, there was a clear difference in the frequency of double, triple and especially quadruple events between the conditions when channels were formed by individual tetramers (1 mM Mg^2+^) and when channels were formed by 2D crystals (10 mM Mg^2+^) (Figure 2g). This indicates that up to four channels within 2D lattices acted in unison. Our density maps of 2D tetramer lattices (Figure 3(giii-gv)) provide a possible mechanism for such coordination: because four tetramers contact each other with the tips of their wings in a tightly packed knot (asterisk in Figure 1b,c), the uncoiling of one tetramer (as described in Figure 3a–f) could cause a shift in the opposing wing in the quaternary complex and lead to respective conformational changes in the diagonally located tetramer. It is conceivable that such changes could occur in a chain of several tetramers, and we have observed coordinated transitions in at least two diagonal tetramers within a 2D crystal.

Third, α-LTX channels seemed to be able to switch between their two open states without closing (Figure 2d,g,h). Again, this transition may simply be too fast for the temporal resolution of our equipment. However, the three-state mechanism of α-LTX channel activity that follows from our observations (Figure 2h) has also been proposed previously [6]. In addition, our 3D reconstructions suggest that both narrow-pore and wide-pore soluble toxin tetramers (Figure 3c,d) possess apparently unrestricted channels through the middle of the tetramer and thus likely represent two different open states of the channel. Nevertheless, the toxin channels are clearly able to close, as has been observed in single-channel recordings both here (Figure 2d–f) and in a wide range of publications [6,19,23]. The mechanism of channel closing is currently unknown, although some of our 3D reconstructions had an axial core of four leg domains below the central channel of the tetramer (Figure 4c) and might represent a closed state of the channel. In contrast, the shift between the narrow and wide pore can now be mechanistically understood as consisting of specific movements of the monomers’ wings, bodies and heads, leading to the widening of the central pore; this increases the diameter of the channel.

The difference in 3D structure between the α-LCT monomers and α-LTX monomers (whether individual molecules or parts of the tetramer) can be explained by a remarkable conformational rearrangement (Figure 4d). This change affects the body domain between AR11 and 12. As a result, the large part of the body domain that arches over the head in α-LCT monomers folds onto itself in αLTX monomers and forms strong contacts both with the upper part of the body and with the head domain. This large curved domain becomes fixed rigidly in a horizontal position, forming the wing domain. A strong interaction between the wing and head domains leads to two consequential changes: (1) the leg domain (Connector domain in α-LCT and δ-LIT), which can no longer occupy the space between the head and body, unfolds and adheres to the lower part of the body, with its end (helices H1-3) protruding on the side of the tetramer (Figure 4(a,b,dii)) and (2) the distorted head domain (e.g., Figure 3c–f) loses α-helices H5 and H6, which move from the head to the bottom of the body, where they fold under the plug domain and AR1-5 of the tetramer (Figure 3c,d and Figure 4b).

So how does the α-LTX tetramer penetrate the membrane? The leg contains a hydrophobic region 1 (α-helix H3, yellow in Figure S1a), which could provide an anchor to interact with the lipid membrane. Furthermore, our reconstructions suggest that the leg domain can detach from the body and form an axial tetrameric core domain, or proboscis, under the center of tetramer (Figure 4c). In our cryoEM reconstructions, this domain anchors the tetramer at the water–air interface and thus is likely to serve as a membrane anchor as well (Figure 4(ei)). However, although the core domain is long enough to pierce the membrane, it probably does not serve as a channel itself, because such an arrangement would leave the bulk of the α-LTX tetramer exposed to solution, while membrane-inserted α-LTX is known to be protected from proteases [35], particularly in the AR area of the body. In addition, as was previously observed by cryoEM, α-LTX tetramers can fully insert themselves into the membrane of liposomes [12], as schematically shown in Figure 4(eii). Therefore, we propose that, after initially anchoring the tetramer in the membrane, the axial core domain brings the tetramer close to the membrane and allows it to interact with the polar heads of lipids. The subsequent retraction of the leg domains, which, while still embedded in the membrane, move away from the center and towards their normal position on the side of the body, causes membrane dilation (Figure 4e). This creates a pore in the lipid bilayer, which soon becomes too wide to be fully bounded by the four hydrophobic α-helices of the leg domains. This likely leads to the fusion of inner and outer bilayer leaflets, forming a hydrophilic toroidal structure (Figure 4(eiii)), as has been shown for many pore-forming toxins [36]. This process is in particular similar to the mechanism of pore formation by actinoporins [37]. As a result, the toxin tetramer, whose outside surface is mostly hydrophilic, becomes surrounded by the membrane, whereas its central pore permeates the membrane. The dimensions of this channel are consistent with the size of the water pore of the α-LTX channel determined by electrophysiological methods, whose cis-side (extracellular) entrance has a diameter of 18 Å [5,38,39].

Finally, another feature of the 2D crystals discovered here—imperfections in the lattice—can make a strong contribution to the biological effect of α-LTX. As shown in Figure S1c,d, gaps with one–four missing tetramers appear during crystal formation. This observation has two important consequences: (1) when the 2D crystals are formed, individual molecules or short strings of tetramers are added to the sides of the lattice, within its plane, and likely cannot be incorporated into the middle of the lattice from above or below and (2) when sheets of α-LTX are incorporated into the membrane, the lipid bilayer may not perfectly fill the gaps in the lattice or may be lost from such gaps due to surface tension, thus creating large holes in the membrane (one missing tetramer creates a gap of ~160 Å in diameter), which must be permeable not only to water and ions but also to most low-molecular-weight solutes. In fact, a number of publications report that α-LTX can cause non-vesicular release by allowing for a leakage of cytoplasmic neurotransmitters and ATP [40,41].

At least some other pore-forming toxins, such as sticholysin from the sea anemones of the *Stichodactyla* genus, can undergo 2D crystallization [37,42,43]. In the presence of lipid monolayers, sticholysin forms 2D monolayer crystals, which consist of cyclical tetrameric units, thus in many ways resembling α-LTX. These findings indicate that 2D crystallization may be an important, albeit little-studied, feature of many pore-forming toxins which contributes to their biological functions. Our results, delineating physiologically relevant structural rearrangements of α-LTX during 2D crystallization and channel transitions, suggest clear avenues of further research into the mechanism of action of latrotoxins.

As this paper was being prepared for publication, an interesting study was published that reported the high-resolution cryoEM structure of α-LTX and the molecular modeling of its pore formation [44]. This work describes how the α-LTX tetramer can anchor itself in the membrane by ejecting from the top of the head a long stalk consisting of the N-terminal α-helices H1-H4 (Connector domain or leg domain), plus helix H5 from the head domain (Figure 4(di)). Similar to the tetrameric axial core domain observed here (Figure 4c,e), but on the opposite side of the tetramer plane, these N-terminal helices H1-H3 might penetrate the lipid bilayer and are proposed to themselves form an ion channel in the membrane. It is interesting to compare this fascinating structure with the α-LTX tetramer described in our work. On the one hand, there is a remarkable similarity of the two top views of the α-LTX tetramer between our and their reconstructions (Figure 3b). On the other hand, there are big differences, in particular in how in our model, the top of the body folds onto itself and how the wing attaches to the body and head. Most importantly, the model described here cannot sustain the release of the stalk domain from above the head domains because the leg domain (Connector domain) is located under the bottom of the tetramer both in the soluble tetramers and in the 2D crystals attached to the water surface (Figure 3 and Figure 4). Furthermore, our tetramer side views (in other words, tetramers in solution and away from the air–water interface) never show any traces of the stalk above the heads, while some of our images demonstrate a structure similar to the stalk (but much shorter) at the bottom of the tetramer, instead (Figure 4c). Also, the gating of the channel formed only by the two helices from each monomer (as in Figure 4(c,ei)) is unclear, and the size of the entrance of such a channel is only 5 Å, inconsistent with physiological data [5,38]. Finally, if the channel is only formed by the membrane insertion of the two helices at the very N-terminus of the toxin, this certainly does not provide protease protection to the rest of the molecule as previously described [35].

What could be the reason for the remarkable similarities and differences between these two models? Notwithstanding the low resolution of our 3D reconstruction, it clearly supports an opposite location of the membrane penetration domains in our model as opposed to the model by Klink and co-authors [44]. As we used the venom from the Central Asian spider *Latrodectus lugubris*, while Klink and co-authors used the venom from the Middle Eastern *L. tredecimguttatus*, it is possible that this disparity may be species-specific. Although this difference may seem too radical, given that the molecules are so similar in many other respects, one needs to keep in mind that the Connector (leg) domain appears to be very flexible and able to assemble into similar tetrameric, proboscis-like structures on either side of the toxin tetramer. Therefore, it is possible that some small differences in the folding of the conservative part of the toxin (AR domains) may force the leg domains to form a proboscis on one or the other side of the tetramer and thus mediate two alternative mechanisms of channel formation that are structurally and functionally similar but topologically distinct.

In summary, our work provides an interesting new look at the mechanisms of pore formation by latrotoxins and at the nature of their toxicity. The ability of α-LTX to form 2D crystals can now be employed for more in-depth studies of this toxin.

## 4. Conclusions

α-LTX forms tetramers which can assemble into rigid 2D crystals. These crystal sheets facilitate the simultaneous formation of ion channels in the cell membrane. Both in the 2D crystals and in single tetramers, α-LTX undergoes conformational changes that increase or decrease the size of the channel pore. Channels of two types with distinct conductivities were observed by electrophysiological recordings, which also indicate that multiple channels formed by 2D crystals can transition between the narrow-pore and wide-pore conformational states.

## 5. Materials and Methods

### 5.1. α-LTX Purification

The toxin was purified from the venom of the Central Asian black widow spider, *Latrodectus lugubris*, using the procedure described previously [13]. Briefly, milked and lyophilized venom was dissolved in buffer A (50 mM Tris-HCl, pH 8.3, 150 mM NaCl), with or without 20 mM EDTA and clarified by centrifugation steps at 14,000× *g*. The supernatant was separated by gel exclusion chromatography on Sephacryl S-400 HR (Amersham Biosciences, Amersham, UK) equilibrated with buffer A. Fractions containing α-LTX (as determined by Western blotting) were separated by ion-exchange chromatography on a Protein-Pak Q 8HR (Waters Corporation, Taunton, MA, USA), with elution by a stepwise gradient of NaCl concentration (0.15, 0.3, 1 M). Fractions containing α-LTX were concentrated and dialyzed by ultrafiltration using a Vivaspin 20 concentrator (10,000 MW cut-off, Sartorius AG, Göttingen, Germany) and then separated by preparative native electrophoresis in a 6% acrylamide gel using Prep Cell model 491 (Bio-Rad Laboratories Ltd., Watford, UK) in 25 mM Tris-HCl, pH 8.3, 192 mM Glycine (Bio-Rad). α-LTX was then concentrated using a Centriprep YM-30 concentrator (30,000 MW cut-off, Merck UK, Feltham, UK) to a final concentration of 0.5 to 1 mg/mL.

### 5.2. Oligomerization Studies

α-LTX was iodinated using Na^125^I by a previously described chloramine T method [16]. Used as a tracer, the 0.1 pM radioactive toxin was mixed with 10 nM native α-LTX and incubated in 20 mM NaCl, 20 mM Tris-HCl, pH8.3 containing 1, 10 or 100 mM MgCl_2_ or 5 mM EDTA, for 7 days. At certain intervals (0 min, 10 min, 30 min, at 20 °C; 1 h, 1 d, 7 d, at 4 °C) aliquots were taken and centrifuged at 14,000× *g*. The radioactivity of pellets and supernatants was determined in a γ-counter. The experiment was repeated 3 times.

### 5.3. CryoEM Specimen Preparation

α-LTX aliquots were diafiltrated to the desired volume and buffer composition using Microcon YM-30 centrifugal filter units (30,000 MW cut-off, Merck). Protein recovery after diafiltration was estimated by SDS-PAGE. Holey carbon-coated 300-mesh copper EM grids (Agar Scientific, Stanstead, UK, or Quantifoil Micro Tools GmbH, Jena, Germany) were glow-discharged in 0.2 mbar air for 15–20 s with a 5 mA current in a TEM Turbo Carbon Coater unit (model 208C, Agar Scientific) and used within 5–100 min. Grids were loaded with 3–4 µL of sample (0.1–1 mg/mL) and plunge-frozen using a controlled-environment freeze-plunging robot (Vitrobot, FEI Company, Hillsboro, OR, USA). The grids were stored in liquid nitrogen and cryo-transferred into the microscope using a cryo-transfer station (model 626.53P50, Gatan, Inc., Pleasanton, CA, USA) and a cryo-specimen holder (model 626, Gatan).

### 5.4. Cryo-Electron Microscopy and Data Collection

Images were collected on a CM-200 cryogenic electron microscope equipped with a field emission gun (Philips Electronics, Eindhoven, The Netherlands), at 50,000× (nominal) magnification under low-dose conditions onto Kodak SO-163 film. Selected negatives were digitized using a patchwork densitometer (Image Science Software GmbH, Berlin, Germany) with a pixel size of 3.02 µm, leading to a pixel size (on the specimen scale) of 1.23 Å.

### 5.5. Image Analysis

Image processing was performed using the IMAGIC-5 [45,46] (versions from circa 2003–2007) and Spider v. 13.00 package [47] software on workstations running UNIX.

The contrast transfer function (CTF) of micrographs was estimated semi-automatically using the TRANSFER program [46] or interactively using the TRANSFER program of Imagic-5. CTF correction was conducted using the TRANSFER program, in FLIP mode, with a single defocus value per patch. Images were then trimmed to 320 × 320 pixels (395 × 395 Å) and coarsened 2-fold (to a pixel size of 2.47 Å). Following CTF correction, images were bandpassed with a low-frequency cut-off of ~165 Å^−1^ and a high-frequency cut-off of ~6 Å^−1^. These parameters were chosen to remove intensity ramps across images. The images were also masked with a circular aperture of ~375 Å diameter, and the average and variance of their densities within this aperture were normalized to 0 and 10, respectively.

Iterative multi-reference alignments against reprojections were carried out using Imagic’s MRALIGN program or the AP NQ program within Spider. MSA analysis and classification was carried out within IMAGIC, using the MSA, CLASSIFY and CLASSUM programs.

For 2D crystal image analysis, micrographs selected on the basis of their diffraction pattern in a laser diffractometer were scanned using a patchwork densitometer (Image Science, Germany). The lattice was indexed, and a mask was computed and applied to the diffraction pattern. The masked pattern was reverse-Fourier-transformed to yield a filtered real-space image. A correlation map between a patch from a highly ordered part of the filtered image and the whole filtered image was computed. The peaks in this correlation map were used to evaluate lattice irregularities, which were corrected by lattice unbending procedures [26]. Finally, all the unit cells were extracted from this filtered, unbent image of the crystal lattice and averaged to give a 2D projection map. To estimate the resolution of this map, the Fourier ring correlation between the averages from two halves of the set of unit cells was computed. Contour maps of 2D images were created using the NPO program of the CCP4 suite [48].

Three-dimensional reconstruction was conducted using exact-filtered back-projection as implemented by the TRUE3D program of Imagic, with the object size set approximately to the maximum radial dimension of the LTX tetramer (from the symmetry axis to the tip of a wing), i.e., ~125 Å. The resolution of 3D reconstructions was estimated by randomly separating images into two groups, calculating a 3D reconstruction from each of the sets of images and calculating the FSC between the two 3D reconstructions [49]. The resolution was estimated as the lowest spatial frequency at which the FSC fell below the ½-bit curve [50].

For 3D statistical (heterogeneity) analysis, 3D reconstructions were each mounted into a single 2D image, by using IMAGIC-5’s MOUNT program, and all mounted reconstructions were collated into a single image stack. MSA was then run for a maximum of 64 iterations, with 69 eigenimages calculated.

Molecules were visualized using UCSF Chimera [51], and figures were prepared in the orthographic projection, with the density threshold for surface rendering set such that the volume would correspond to the molecular weight of the LTX tetramer (520 kDa).

### 5.6. Cell Culture and Electrophysiology

We used human embryonic kidney 293 (HEK293) stably expressing a mutant receptor, which contained the extracellular N-terminal domain of ADGRL1/latrophilin-1 and the first transmembrane domain [31]. The cells were grown in Dulbecco’s modified Eagle’s medium containing 10% heat-inactivated fetal bovine serum, 100 mg/mL penicillin and 100 mg/mL streptomycin (Gibco, Thermo Fisher Scientific, Inc., Waltham, MA, USA). A few hours prior to experiments, the cells were dislodged from plates using the recording solution supplemented with 5 mM EDTA and replated at 50,000 cells/well into multi-well plates containing 25 mm glass coverslips coated with poly(L-lysine). In some experiments, the medium contained 100 μM cytarabine to inhibit cell division. About 2 h later, the coverslips were transferred into a heated recording chamber (Warner Instruments, Holliston, MA, USA) and observed under an inverted fluorescent microscope TE200 (Nikon Corporation, Tokyo, Japan) equipped with a color video camera and a motorized microscope stage/focus. Cells were constantly perfused with the extracellular recording solution containing required ions at a flow rate of 1 mL/min, except during the addition of α-LTX (as shown in Figure 2).

Whole-cell currents were recorded on isolated cells in an extracellular solution containing (in mM) 135 NaCl, 3.5 KCl, 1 MgCl_2_, 2 CaCl_2_, 10 HEPES, 10 glucose and 1 mg/mL bovine serum albumin; pH 7.4. For Ca^2+^-free recordings, 2 mM CaCl_2_ was replaced with 1.5 mM EGTA-Na_4_; for high Mg treatment, the extracellular solution contained 10 mM MgCl_2_ and 123 mM NaCl. The 5–10 MΩ patch pipettes were prepared using a Model P2000 puller (Sutter Instrument Company, Novato, CA, USA) from borosilicate glass capillaries (Harvard Apparatus, Holliston, MA, USA) and filled with an intracellular solution consisting of the following (in mM): 145 CsCl, 1 MgCl_2_, 10 HEPES, 2 Mg-ATP and 0.2 GTP-Na2; pH 7.2. Once the voltage clamp conditions were established, the extracellular buffer was replaced with a calcium-free solution containing the required Mg^2+^ concentration. At the end of some low-Mg experiments, the chamber was perfused with the high-Mg buffer, and the recording continued for ~1 min. For recording α-LTX-induced currents in outside-out membrane patches, the pipettes were coated with Sylgard (Dow Corning Corporation, Midland, MI, USA) and heat-polished. Cells and membrane patches were voltage-clamped at a holding potential of –60 mV, using a Model 2400 patch-clamp amplifier (A-M Systems, Inc., Sequim, WA, USA controlled by the pClamp 11 software (Axon Instruments, Molecular Devices, LLC, San Jose, CA, USA). After first α-LTX channels were detected, the chamber was flushed for 5 min; then, perfusion was stopped again during I_m_ recordings. Membrane currents were filtered at 2 kHz using an LPF202A filter/amplifier (Warner Instruments, USA) and a HumBug harmonic frequency quencher (Quest Scientific, Digitimer, Welwyn Garden City, UK) and then digitized at 10 kHz using a Digidata 1322A digitizer (Axon Instruments, USA). The records were visually inspected for irregularities, lowpass-filtered at 4–8 kHz and idealized using the open source clampSeg software version 1.1-1 [52], with an empirical selection of the idealization method; gating events were interactively detected and normalized. 

The normally distributed electrophysiological data were compared using a *t* test. The null hypothesis was rejected at *p* < 0.05; the following indicators of probability were used in the paper: **, *p* < 0.01; ***, *p* < 0.001.

## Data Availability

Data are contained within the article.

## Supplementary Materials

The following supporting information can be downloaded at https://www.mdpi.com/article/10.3390/toxins16060248/s1, Figure S1: α-LTX crystallization conditions and parameters.; Figure S2: Different conformations of the toxin tetramer in 3D reconstruction obtained by single particle analysis.
