# Supplementary material for: α-Latrotoxin Tetramers Spontaneously Form Two-Dimensional Crystals in Solution and Coordinated Multi-Pore Assemblies in Biological Membranes
Source: Toxins (Basel). 2024 May 27;16(6):248. doi: 10.3390/toxins16060248 (PMC11209280; doi:10.3390/toxins16060248)
Supplement: Supplementary file 1 [file toxins-16-00248-s001.zip › toxins-2965184-supplementary.pdf]

Supplementary Materials: α-Latrotoxin Tetramers Spontaneously Form Two-Dimensional Crystals in Solution and Coordinated Multi-pore Assemblies in Biological Membranes

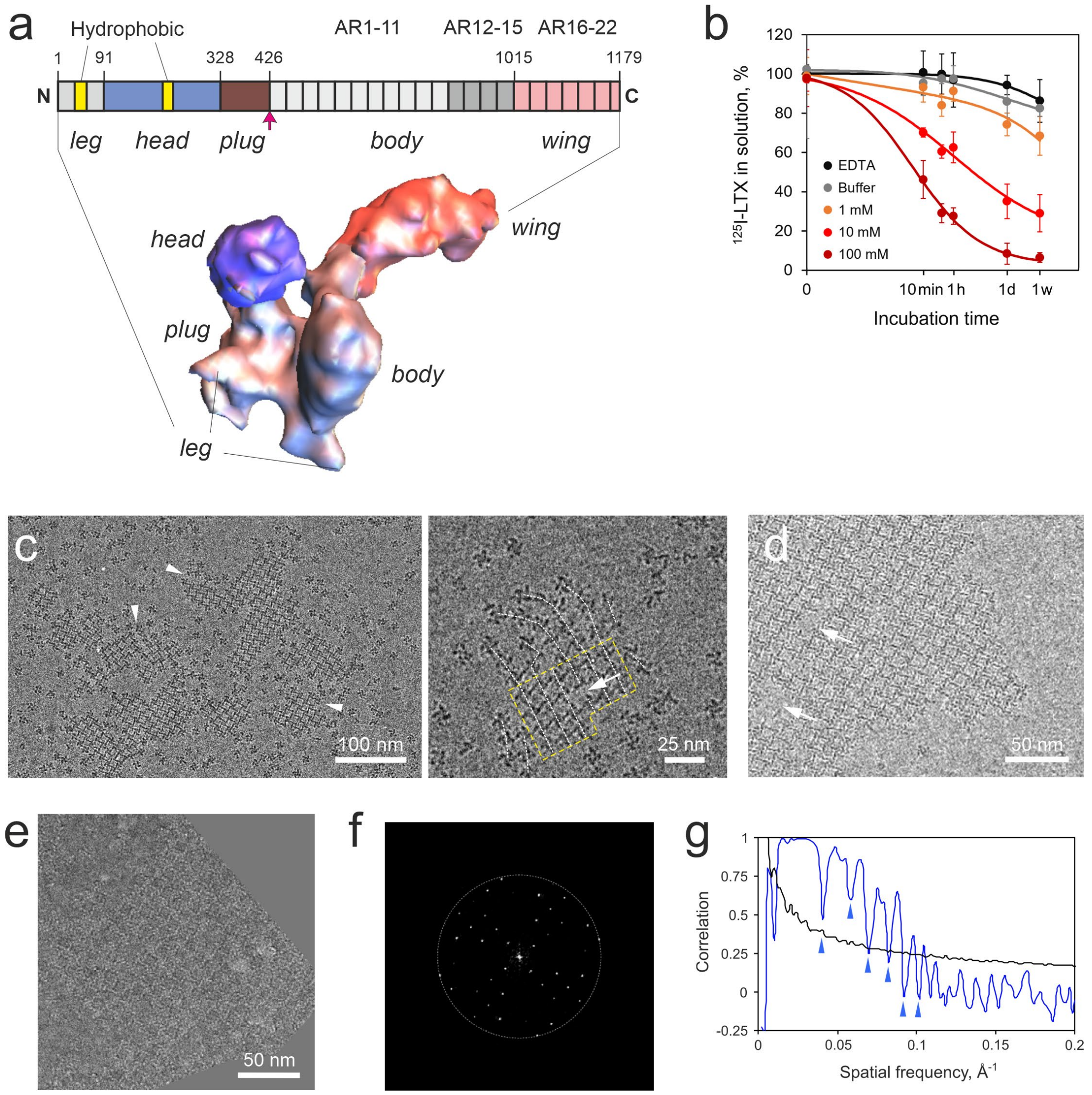

Figure S1. α-LTX crystallization conditions and parameters. (**a**) The domain structure of α-LTX. Top, a block diagram of the toxin sequence. Bottom, a 3D model of toxin monomer extracted from a 3D reconstruction (corrected based on the crystal data in Figure 1 and on the cryoEM structures of α-LCT and δ-LIT [25]). Grey and pink rectangles are ARs. Amino acid residue numbers are shown above the protein sequence diagram. The α-LTX domains are listed on both the sequence diagram and the 3D structure: *head*, *body*, *wing*, *leg*. The purple arrows indicate the position of insert in the mutant LTX^{N4C} [59]. (**b**) The formation of supramolecular assemblies of α-LTX in the presence different concentrations of Mg^{2+}. Radioactively labelled 10 nM α-LTX was incubated in buffer containing 0-100 mM Mg^{2+} or 5 mM EDTA (as shown on the graph) and centrifuged at the indicated intervals. Note the logarithmic scale of the time axis. A decrease in soluble radioactivity indicates the presence of large (precipitable) molecular species. (**c**) Examples of multiple growing 2D crystals produced by 800 nM α-LTX incubated with 10 mM Mg^{2+} for 20 min. The micrograph was obtained at 7.3 μm defocus. Protein density is in black. Left, multiple growing crystal nuclei. The arrowheads point at strings of tetramers attaching to the compact nuclei. Right, a growing 2D crystal nucleus. The white lines indicate tetramer strings constituting the compact nucleus (yellow line) and continuing outside it; short strings of tetramers are seen attaching to the sides

of the nucleus. The arrow indicates a one-tetramer hole in the compact lattice. (**d**) Large crystals are formed by 6 μM α-LTX incubated in 10 mM Mg^{2+} for 5-10 s. The micrograph was obtained at 2.33 μm underfocus. Protein density is in black. The arrows show holes in the lattice. (**e**) A micrograph of the 2D crystal used to produce the projection map in Figure 1b. Protein density is in white. (**f**) A power spectrum of the 2D crystal used to obtain the projection map shown in Figure 1b. The dashed circle: 32 $Å^{-1}$. (**g**) A Fourier ring correlation (FRC) between two projection maps, each calculated from one half of the set of unit cells. The black curve: 3σ threshold. The arrowheads indicate the positions of the first six zeroes in the predicted phase contrast transfer function (CTF) at 2.33 μm, which coincide with low-correlation spatial frequencies.

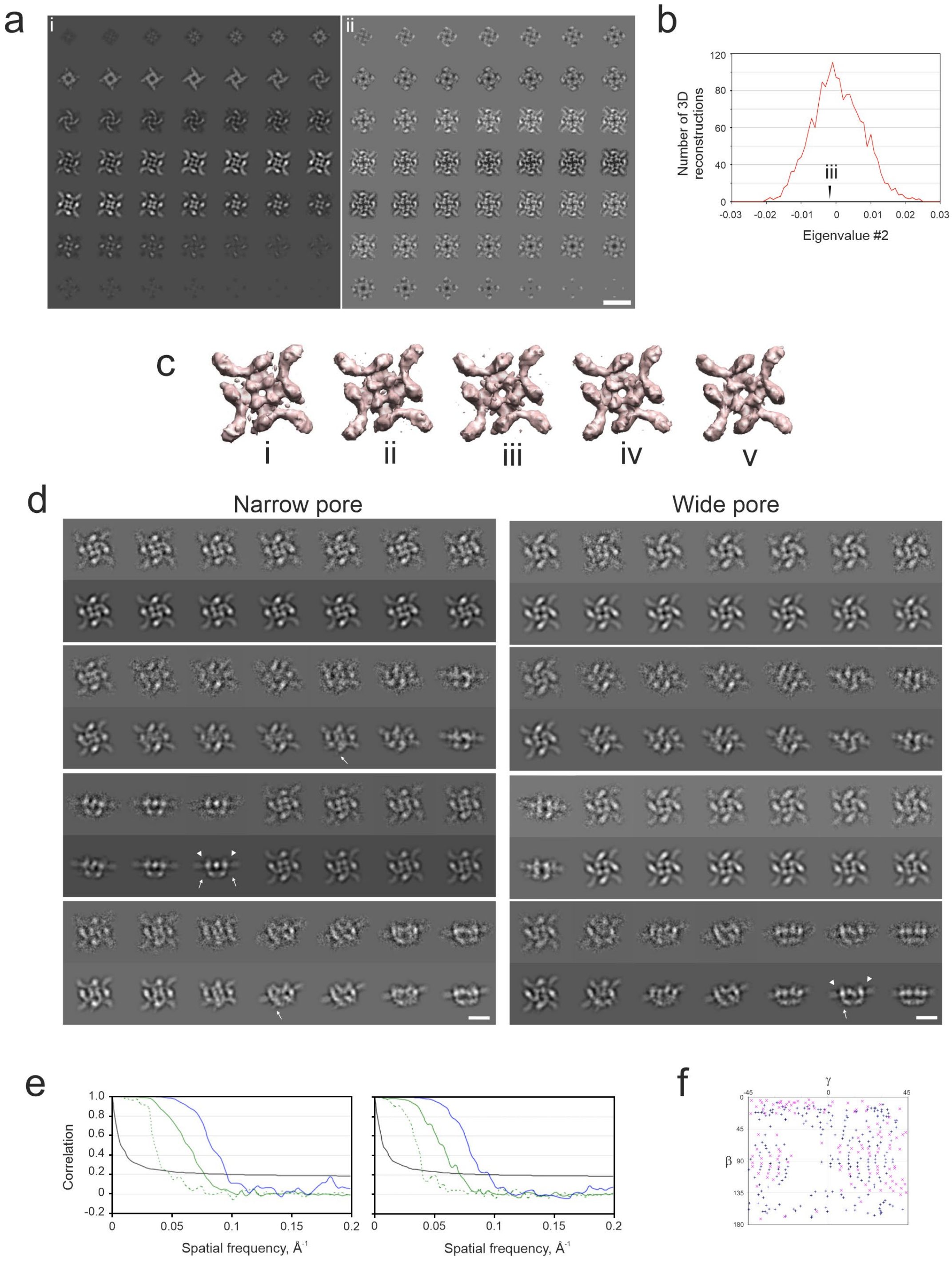

Figure S2. Different conformations of the toxin tetramer in 3D reconstruction obtained by single particle analysis. (**a**) Three-dimensional eigenvolumes computed from a set of 2,500 3D reconstructions, viewed from the bottom of the tetramer. The reconstructions were calculated using back projection into the C4 asymmetric triangle, from sets of 25 average images randomly selected from a set of 344. The first two eigenimages calculated by MSA of the set of 3D reconstruction are shown as mounted slices through the 3D reconstruction volume. Scale bar: 20 nm. (**b, c**) A visual inspection of the 3D reconstructions with varying eigenvalue #2. (**b**) A histogram of eigenvalue

#2, associated with eigenvolume 2 shown in (**a**). (**c**) Surface representations of 3D reconstructions with the following eigenvalues: (i) the lowest, (ii) second lowest, (iii) 1043rd highest, (iv) second highest, and (v) highest, with identical calculated molecular masses. The position of 3D reconstruction (iii) is shown in the histogram, the arrowhead in (**b**). The central pore diameters varied from 11 Å (i) to 12.7 Å (iii) to 13.5 Å (v). (**d**) Randomly selected average images and reprojections of the narrow- and wide-pore 3D models at different angles. The arrowheads indicate the lower position of the wing relative to the top of the body domain (compared to the α-LTX reconstruction in [49]); the arrows point at the leg domain protruding from the low side of the body. (**e**) (i, ii) Resolution estimates of the narrow- and wide-pore 3D reconstructions. Fourier shell correlations (FSC) between the two 3D reconstructions calculated from half of the average images used in the narrow-pore (i) and wide-pore (ii) 3D reconstructions. The dashed curve, the FSC before refinement; the solid green curve, the FSC after four iterations of refinement; the solid blue curve, the FSC after four iterations of refinement and masking (low-pass filtered in real-space with a ~25-Å kernel). (i) The resolution of the narrow-pore reconstruction was ~22 Å before refinement, and 10.5 Å after refinement and masking (½-bit threshold). (ii) The resolution of the wide-pore reconstruction was ~25 Å before refinement, and 10.5 Å after refinement and masking (½-bit threshold). (**f**) The distribution of narrow-pore (pink) and wide-pore (blue) projections on the C4 asymmetric triangle. The β and γ Euler angles define projection directions assigned to the average images used in the 3D reconstructions.
